# Dapagliflozin attenuates atrial fibrosis via the HMGB1/RAGE pathway in atrial fibrillation rats

**DOI:** 10.1515/biol-2025-1163

**Published:** 2025-09-08

**Authors:** Zhenni Tan, Jianxiang Chang, Yin Li, Xiang Sun, Fanxiang Liu, Yang Chen, Lin Pan

**Affiliations:** Department of Cardiology, The First Affiliated Hospital of Shaoyang University, Shaoyang, 422000, Hunan, China; Department of Emergency, The Central Hospital of Shaoyang, Shaoyang, 422000, Hunan, China

**Keywords:** HMGB1/RAGE pathway, DAPA, atrial fibrillation rats, myocardial fibrosis

## Abstract

Atrial fibrillation (AF) is the most prevalent sustained cardiac arrhythmia. A key pathological feature of AF is atrial fibrosis, which promotes arrhythmogenic remodeling. While myocardial fibrosis has been widely observed in AF models, the underlying molecular mechanisms driving fibrotic progression remain incompletely understood. AF rats were modeled using acetylcholine, followed by treatment with different concentrations of dapagliflozin (DAPA) or positive control amiodarone. To elucidate the role of the high-mobility group box 1 (HMGB1)/receptor for advanced glycation end products (RAGE) pathway in AF, lipopolysaccharide (LPS; an HMGB1/RAGE pathway activator) and FPS-ZM1 (a RAGE inhibitor) were employed. Cardiac function, myocardial fibrosis, and inflammation-related proteins were assessed using echocardiography, enzyme-linked immunosorbent assay, histological staining, Western blotting, and reverse transcription quantitative polymerase chain reaction. AF rats exhibited marked cardiac dysfunction, fibrosis, and increased expression of inflammatory markers. DAPA restored cardiac function, attenuating fibrosis and inflammation. LPS aggravated cardiac injury, while DAPA attenuated the damage, with the greatest protective effects observed in the LPS + DAPA + FPS-ZM1 group. DAPA attenuates atrial fibrosis and cardiac dysfunction in AF rats by inhibiting the HMGB1/RAGE pathway. This study suggests the potential of DAPA as a therapeutic option for AF.

## 1 Introduction

Atrial fibrillation (AF) ranks among the most prevalent sustained cardiac arrhythmias encountered in clinical practice and is associated with increased morbidity, mortality, and healthcare burden worldwide [1,2]. Under normal physiological conditions, the atria contract in a regular manner to ensure effective pumping function of the heart [3,4]. However, in patients with AF, rapid and disorganized atrial electrical activity impairs atrial contraction, leading to reduced cardiac efficiency, thromboembolic events, stroke, heart failure, and myocardial ischemia [5,6]. These complications greatly impact patient prognosis and quality of life. Although catheter ablation remains a cornerstone of AF treatment, its high cost and recurrence rate limit broad applicability [7], underscoring the urgent need for novel, safe, and effective therapeutic strategies.

Atrial fibrosis, characterized by excessive extracellular matrix deposition and structural remodeling of the atrial myocardium, is a hallmark of atrial cardiomyopathy and plays a pivotal role in the initiation and maintenance of AF [8]. Meanwhile, atrial remodeling, particularly structural remodeling driven by myocardial fibrosis, is a key contributor to the progression of AF [9]. Myocardial fibrosis refers to the accumulation of fibrotic tissue within the heart, which disrupts normal atrial structure and function [10]. This process is primarily mediated by activated cardiac fibroblasts that differentiate into myofibroblasts, producing large quantities of collagen and fibrotic proteins [11]. Targeting the pathways that regulate fibroblast activation and fibrotic protein synthesis has therefore become a key therapeutic focus in efforts to mitigate atrial remodeling in AF.

Dapagliflozin (DAPA), a sodium-glucose co-transporter 2 (SGLT2) inhibitor, was initially developed for the treatment of diabetes [12,13]. Research suggests that DAPA can alleviate cardiac fibrosis and inflammation by inhibiting multiple inflammatory signaling pathways, thereby improving cardiac function [14,15]. Notably, recent studies have shown that DAPA is capable of regulating the high mobility group box 1 (HMGB1) signaling pathway in various disease models, including kidney and inflammatory disorders [16,17]. HMGB1, normally a nuclear protein, is released extracellularly following cellular stress or injury. Binding of HMGB1 to receptor for advanced glycation end products (RAGE) triggers pro-inflammatory and pro-fibrotic signaling cascades, contributing to cardiac remodeling and fibrosis [18,19]. The HMGB1/RAGE signaling pathway has garnered increasing attention as a damage-associated molecular pattern (DAMP) pathway [20,21]. However, the potential role of DAPA in mitigating AF-related myocardial fibrosis via HMGB1/RAGE signaling remains insufficiently defined.

This study aims to investigate whether DAPA attenuates myocardial fibrosis in AF rats by modulating the HMGB1/RAGE signaling pathway. The findings may provide mechanistic insights and support the therapeutic potential of DAPA in the management of AF.

## 2 Materials and methods

### 2.1 Animal experiment

A total of 54 male Sprague-Dawley rats (8 weeks old, 230–250 g) were purchased from Slack Jingda Laboratory Animal (Changsha, Hunan, China). All animals were kept in specific pathogen-free environment with temperature of 20–22°C, a 12-h light–dark cycle, and *ad libitum* access to food and water. After a 7-day acclimatization period, 48 rats were randomly chosen to establish AF models by tail vein injection of acetylcholine (60 μg/mL, 1 mL/kg; B50001, Wuyejia Technology, Beijing, China) and calcium chloride (10 mg/mL) once daily for 7 consecutive days [22]. The remaining 6 rats received saline (1 mL/kg) daily for the same duration and served as the control group.

Rats were allocated randomly into nine groups (*n* = 6/group): (1) control group; (2) AF group; (3) DAPA-L group (low-dose DAPA, 0.5 mg/kg, administered via oral gavage); (4) DAPA-M group (medium-dose DAPA, 1 mg/kg, administered via oral gavage) [23]; (5) DAPA-H group (high-dose DAPA, 2 mg/kg, administered via oral gavage); (6) amiodarone (AMIO) group (positive control for AF treatment, 50 mg/kg, administered via oral gavage) [24]; (7) lipopolysaccharide (LPS) group (HMGB1 agonist, 1 mg/mL, administered intraperitoneally) [25]; (8) LPS + DAPA group (2 mg/kg DAPA by gavage + 1 mg/mL LPS, administered intraperitoneally); and (9) LPS + DAPA + FPS-ZM1 group (2 mg/kg DAPA, administered via oral gavage + 1 mg/mL LPS administered intraperitoneally + 1 mg/mL FPS-ZM1, a RAGE inhibitor, administered intraperitoneally.

**Figure 1:**
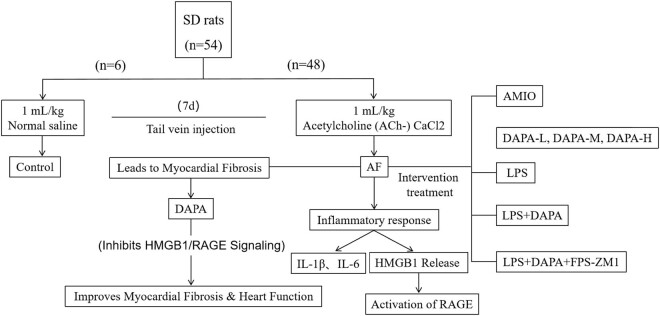
Schematic representation of the animal experimental design.


At the end of the experiment, AF induction was confirmed using electrocardiogram. Rats were euthanized via intraperitoneal injection of 2% sodium pentobarbital (150 mg/kg). Hearts were harvested, washed with phosphate buffer saline, and stored at −80°C for further analysis. The experimental process is shown in Figure 1.

**Ethical approval:** The research related to animal use has been complied with all the relevant national regulations and institutional policies for the care and use of animals and has been approved by the Ethics Committee of The First Affiliated Hospital of Shaoyang University (SY20241131).

### 2.2 Blood glucose (Glu) measurement

Fasting Glu was measured in rats from each group using a glucometer (Contour Plus, Bayer Healthcare, Germany). Blood samples were collected from the tail vein of the rats following overnight fasting. Glu concentrations were determined according to the manufacturer’s instructions.

### 2.3 Electrocardiography (ECG)

ECG was recorded using the FX8322 system (Fukuda Denshi, Japan). Signals were filtered (0.5–150 Hz), amplified (20 mm/mV), and recorded at a paper speed of 50 mm/s. For baseline ECG recordings, a paper speed of 25–100 mm/s and an amplitude of 1 mm/mV were used.

### 2.4 Ultrasonic chocardiography analysis

Under isoflurane anesthesia, rats were subjected to echocardiographic examinations. Transthoracic ECG was performed using a Doppler ultrasound system. Images were obtained and analyzed using the VisualSonics Vevo 2100 imaging system (VisualSonics Inc., Toronto, Canada). Parameters included left ventricular end-systolic diameter (LVESD), left ventricular end-diastolic diameter (LVEDD), left atrial diameter (LAD), left ventricular ejection fraction (LVEF), and left ventricular fractional shortening (LVFS) were tested. Data were averaged over three cardiac cycles for accuracy.

### 2.5 Assay for biomarkers of myocardial injury

Blood samples were obtained from the rat abdominal aorta and allowed to clot for 1 h at room temperature. Serum was separated by centrifugation at 3,500 × *g* for 15 min, and the resulting supernatant was collected. The levels of serum cardiac troponin I (cTnI, ml059111, Shanghai Enzyme-Linked Biotechnology Co., Ltd), creatine kinase-MB (CK-MB, D731144, Shanghai Sangon Biotech Co., Ltd), lactate dehydrogenase (LDH, ml106660, Shanghai Enzyme-Linked Biotechnology Co., Ltd), atrial natriuretic peptide (ANP, ml106992, Shanghai Enzyme-Linked Biotechnology Co., Ltd), and N-terminal pro–brain natriuretic peptide (NT-Pro BNP, D731098, Shanghai Sangon Biotech Co., Ltd) were measured using an automated biochemical analyzer (BS-240VET, Mindray, Shenzhen, China).

### 2.6 Westen blotting (WB)

Tissue samples were homogenized in RIPA lysis buffer (P0013B, Beyotime, Shanghai, China) supplemented with protease inhibitors. The protein concentration of each lysate was determined using a BCA protein assay kit (A55860, Thermo Scientific, Shanghai, China) according to the manufacturer’s instructions. Equal amounts of proteins (20 μg per lane) were loaded onto 10% sodium dodecyl sulfate polyacrylamide gel electrophoresis and subsequently transferred to a polyvinylidene fluoride membrane. After blocking with 5% non-fat milk in tris-buffered saline with Tween-20 (TBST) for 1 h at room temperature, the membranes were incubated overnight at 4°C with the following primary antibodies: HMGB1 (1:1,000, A2553; ABclonal, Wuhan, China), RAGE (1:1,000, A23422; ABclonal), IL-1β (1:1,000, A1112; ABclonal), TNF-α (1:1,000, A11534; ABclonal), IL-6 (1:1,000, A0286; ABclonal), and glyceraldehyde-3-phosphate dehydrogenase (GAPDH) (1:1,000, ab8245, Abcam, UK) as an internal control. The membranes were washed three times with TBST and then incubated with a horseradish peroxidase-conjugated secondary antibody (1:1,000, AS014; ABclonal) at room temperature for 2 h. Protein bands were visualized using an enhanced chemiluminescence detection system (Amersham Pharmacia, Sweden) and quantified by ImageJ software (NIH, USA).

### 2.7 Reverse transcription quantitative polymerase chain reaction (RT-qPCR)

Total RNA was extracted using TRIzol reagent kit (Invitrogen, Carlsbad, CA, USA), and mRNA was converted to cDNA using the EasyScript First-Strand cDNA Synthesis SuperMix (AE301-02, TransGen Biotech). PCR amplification was carried out with the SYBR Green I fluorochrome Pro Taq HS premix qPCR kit (AG11756, Accurate Biotechnology, Changsha, China) following the manufacturer’s guidelines. RT-qPCR analysis was performed using the Applied Biosystems (Foster City, CA, USA) QuantStudio 5 Real-Time PCR system. GAPDH served as the internal control, and the relative expression levels were determined using the 2^−ΔΔCt^ method. Each experiment was performed in triplicate. The primer sequences are shown in Table 1.

**Table 1:** Primer sequences

Gene	Forward primer	Reverse primer
HMGB1	AGTGAGGGAGAGAGTGGGTAA	GAACACTACAGCCTGCCGAG
RAGE	GGGTCACAGAAACCGGTGAT	ATCATGTGGGCTCTGGTTGG
IL-1β	AGCTTCAGGAAGGCAGTGTC	TCAGACAGCACGAGGCATTT
IL-6	CCAGTTGCCTTCTTGGGACT	CTGGTCTGTTGTGGGTGGTA
GAPDH	GACAGTCAGCCGCATCTTCT	GCGCCCAATACGACCAAATC

### 2.8 Hematoxylin and eosin (HE) staining

HE staining was used to assess the histological structure and pathological injury of the atrial tissue. Left atrial tissues were fixed in 4% paraformaldehyde (P0099, Beyotime, Shanghai, China), dehydrated using gradient ethanol, paraffin-embedded, sectioned at 4 μm, and stained using HE staining (Sigma Aldrich, St. Louis, MO, USA). The stained sections were observed using a light microscope (Leica Microsystems, Wetzlar, Germany).

### 2.9 Masson’s trichrome staining

Masson’s trichrome staining was performed to evaluate myocardial fibrosis (collagen deposition) in the atrial tissue. Paraffin-embedded left atrial tissues (4 μm) were subjected to Masson’s trichrome staining, followed by examination under an optical microscope (CKX41, Olympus, Tokyo, Japan) at 400× magnification. Collagen volume fraction (CVF) was quantified using ImageJ software in three random fields per section.

### 2.10 Immunohistochemistry (IHC)

IHC staining for COLI, FGF-2, and α-SMA was conducted on left atrial tissue sections to identify fibrosis-related protein expression. Briefly, samples were fixed, paraffin-embedded, and sectioned into 4 μm thickness. Sections were incubated overnight at 4°C with primary antibodies against COLI (1:1,000, ab270993; Abcam, UK), FGF-2 (1:200, A3908; ABclonal), and α-SMA (1:200, A2235; ABclonal), followed by incubation with a goat anti-rabbit secondary antibody (1:200, AS014; ABclonal) at room temperature for 2 h. DAB (ZY6SK2020; Zeye-BIO, Shanghai, China) was used for visualization and hematoxylin for counterstaining. Staining was observed under a microscope (Nikon, Tokyo, Japan) and analysis using an ImageJ analysis software.

### 2.11 Statistical analysis

Statistical analyses were performed using GraphPad Prism 9 (Dotmatics, Boston, MA, USA). All data are presented as mean ± standard deviation. Comparisons between two groups were performed using a *t*-test, whereas one-way analysis of variance with Tukey’s *post hoc* test was utilized for multiple group comparisons. A *p-*value of less than 0.05 was considered as statistically significant.

## 3 Results

### 3.1 DAPA restores cardiac function in AF rats

**Figure 2:**
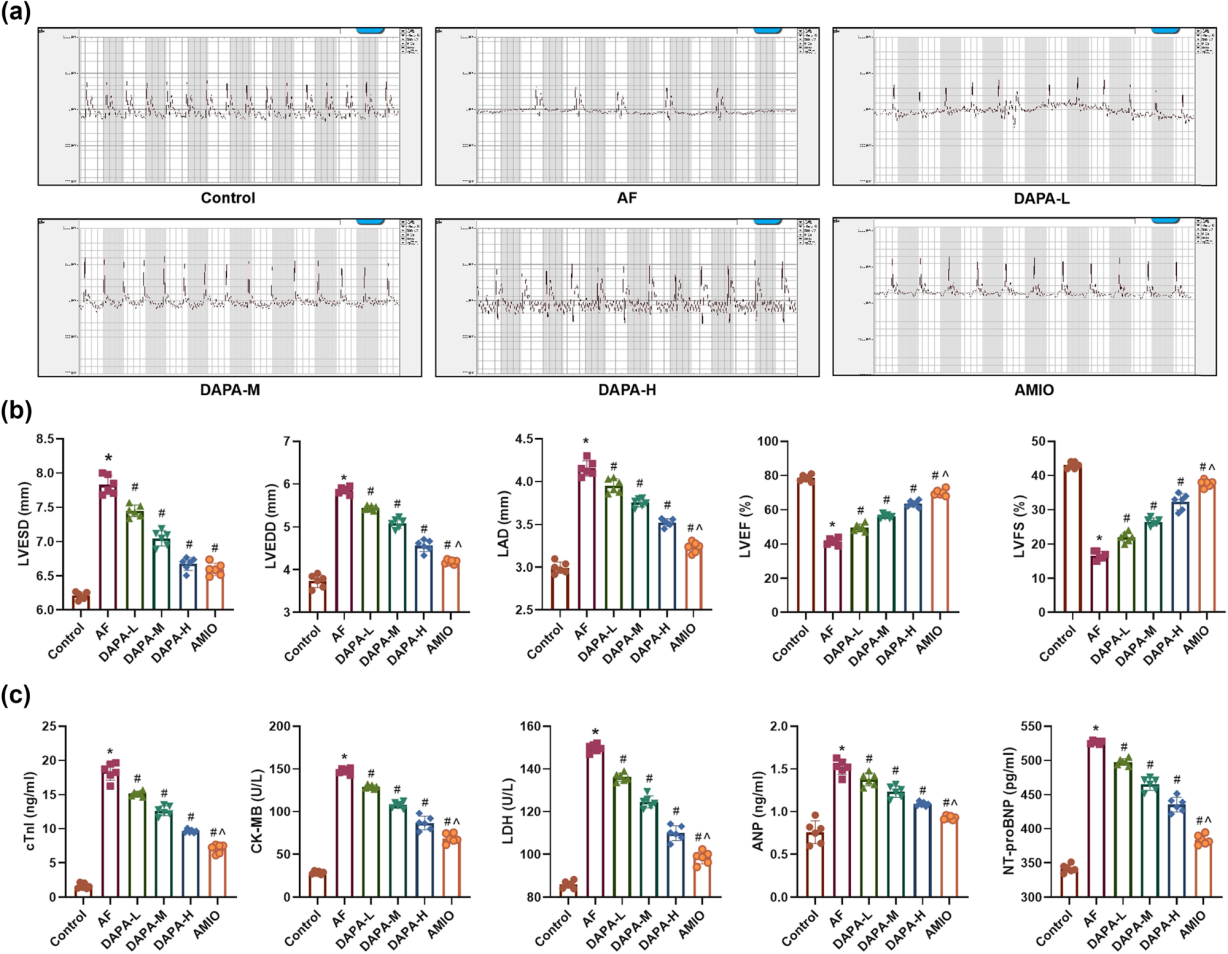
Effects of DAPA on restoring cardiac function in AF rats. (a) ECG assessment of AF in rats. (b) Echocardiographic measurements of cardiac function parameters in AF rats (LVESV, LVEDV, LAD, LVEF, LVFS). (c) Biochemical analysis of myocardial injury markers (cTnI, CK-MB, LDH, ANP, NT-pro BNP) in left atrium tissue from AF rats. *n* = 6. *p < 0.05 vs control, ^#^ *p* < 0.05 vs AF, ^*p* < 0.05 vs DAPA-H.

Glu levels did not differ significantly among the Control, AF, DAPA-L, DAPA-M, DAPA-H, and AMIO groups. Although Glu concentrations showed a downward trend as DAPA concentrations increased, the differences were not statistically significant (*p* > 0.05). ECG confirmed successful induction of AF in rats, as rats in the AF group exhibited P wave disappearance, irregular rhythm, and unstable R–R intervals, whereas rats in the Control group displayed regular P waves and a stable sinus rhythm. DAPA treatment led to a dose-dependent suppression of arrhythmic changes, with the DAPA-H group showing the most significant improvement (Figure 2a). Regarding cardiac function, the AF group showed significantly elevated levels of LVESD, LVEDD and LAD, while LVEF and LVFS were markedly decreased (Figure 2b, *p* < 0.05). Treatment with DAPA (all doses) significantly reduced LVESD, LVEDD, and LAD and improved LVEF and LVFS compared to the AF group, with the most notable effects in the DAPA-H group (*p* < 0.05). Additionally, the AMIO group demonstrated further enhancement in cardiac function compared to the DAPA-H group (*p* < 0.05). Biomarkers of cardiac injury (cTnT, CK-MB, ANP, and NT-proBNP) were significantly elevated in the AF group (Figure 2c, *p* < 0.05). DAPA treatment significantly reduced the levels of these markers in a dose-dependent manner, with the DAPA-H group showing the greatest reduction. The AMIO group displayed even lower levels of these biomarkers than the DAPA-H group (*p* < 0.05).

### 3.2 DAPA alleviates myocardial fibrosis in AF rats

**Figure 3:**
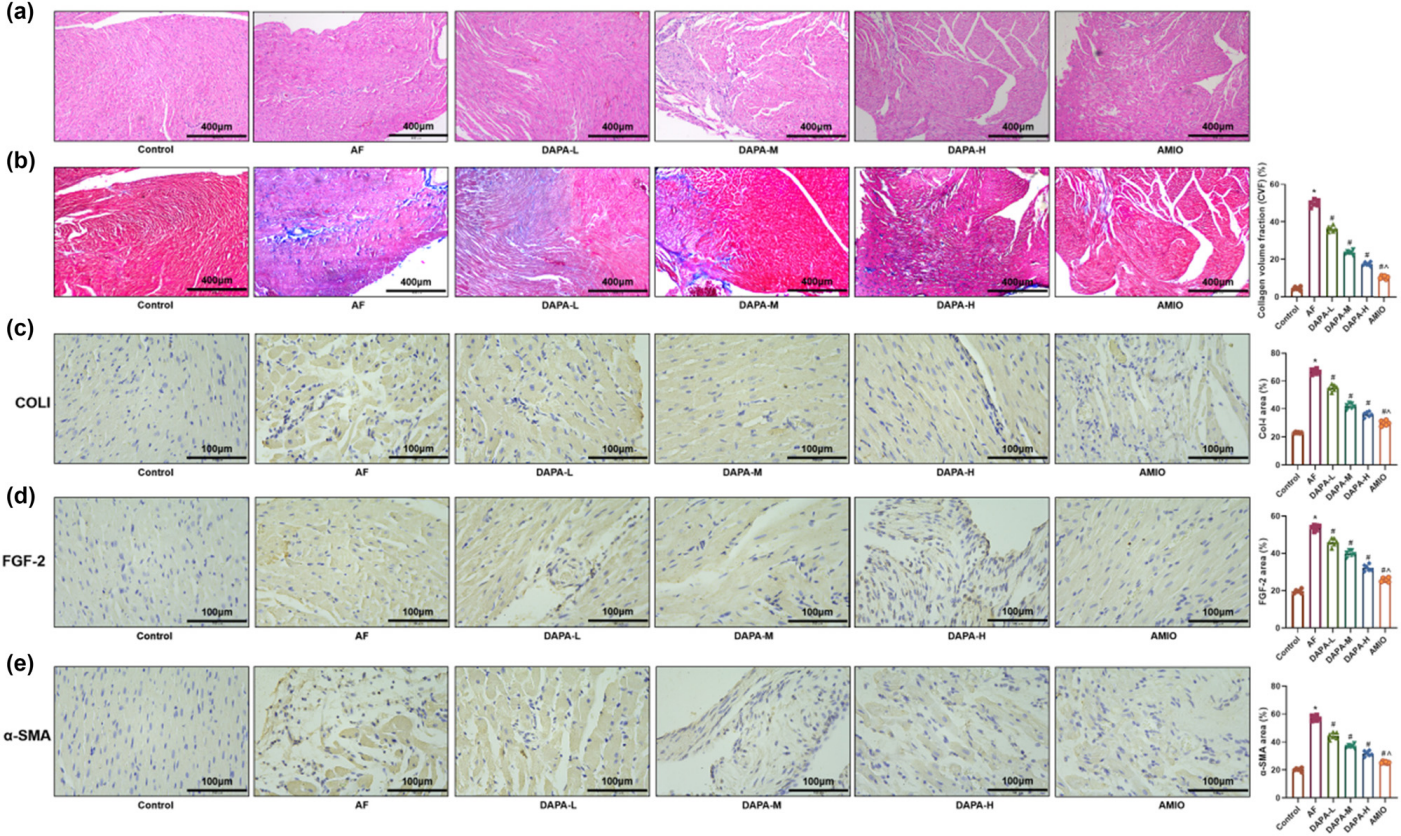
DAPA alleviates myocardial fibrosis in AF rats. (a) HE staining evaluating pathological damage in the left atrial tissue of AF rats. (b) Masson’s trichrome staining assessing myocardial fibrosis in the left atrium of AF rats. (c–e) IHC staining evaluating the expression of COL1, FGF-2, and α-SMA in left atrial tissue. *n* = 6. *p < 0.05 vs control. ^#^ *p* < 0.05 vs AF. ^*p* < 0.05 vs DAPA-H.

HE staining revealed that atrial tissues in the AF group exhibited irregular cell morphology, disordered arrangement, increased interstitial cells, and notable collagen deposition. Additionally, the CVF% was significantly increased, and elevated expression of fibrosis-related proteins COLI, FGF-2, and α-SMA was observed in the AF group (Figure 3a and b, *p* < 0.05). DAPA treatment led to dose-dependent improvements in myocardial architecture, with more regular morphology, reduced interstitial cells, and diminished fibrosis. The expression of fibrosis-related markers was significantly reduced in all DAPA-treated groups, especially in the DAPA-H group (Figure 3c–e, *p* < 0.05). AMIO further attenuated myocardial fibrosis compared to the DAPA-H group (*p* < 0.05).

### 3.3 DAPA curtails the activity of HMGB1/RAGE pathway

**Figure 4:**
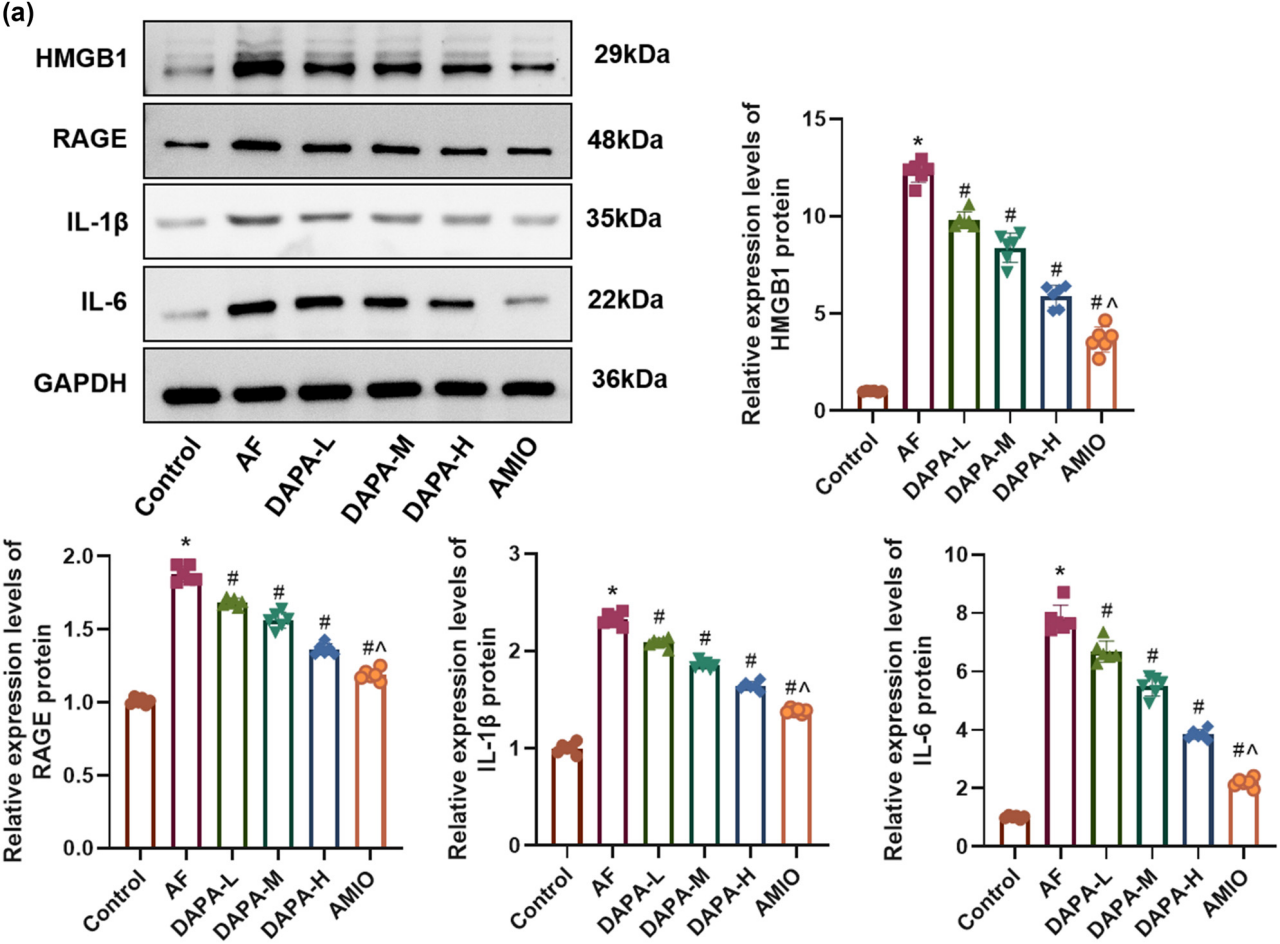
DAPA suppresses the activity of the HMGB1/RAGE inflammatory pathway. (a) WB analysis of the expression levels of HMGB1, RAGE, IL-1β, and IL-6 in left atrial tissues. *n* = 6. *p < 0.05 vs control. ^#^ *p* < 0.05 vs AF. ^*p* < 0.05 vs DAPA-H.

The AF group exhibited significantly elevated levels of HMGB1, RAGE, IL-1β, and IL-6 compared to the control group (*p* < 0.05). DAPA administration resulted in a dose-dependent reduction in these inflammatory markers, with the DAPA-H group showing the most pronounced effect (Figure 4a, *p* < 0.05). AMIO also suppressed HMGB1/RAGE pathway-related proteins more effectively than DAPA-H.

### 3.4 HMGB1/RAGE pathway contributes to the cardioprotective effects of DAPA

**Figure 5:**
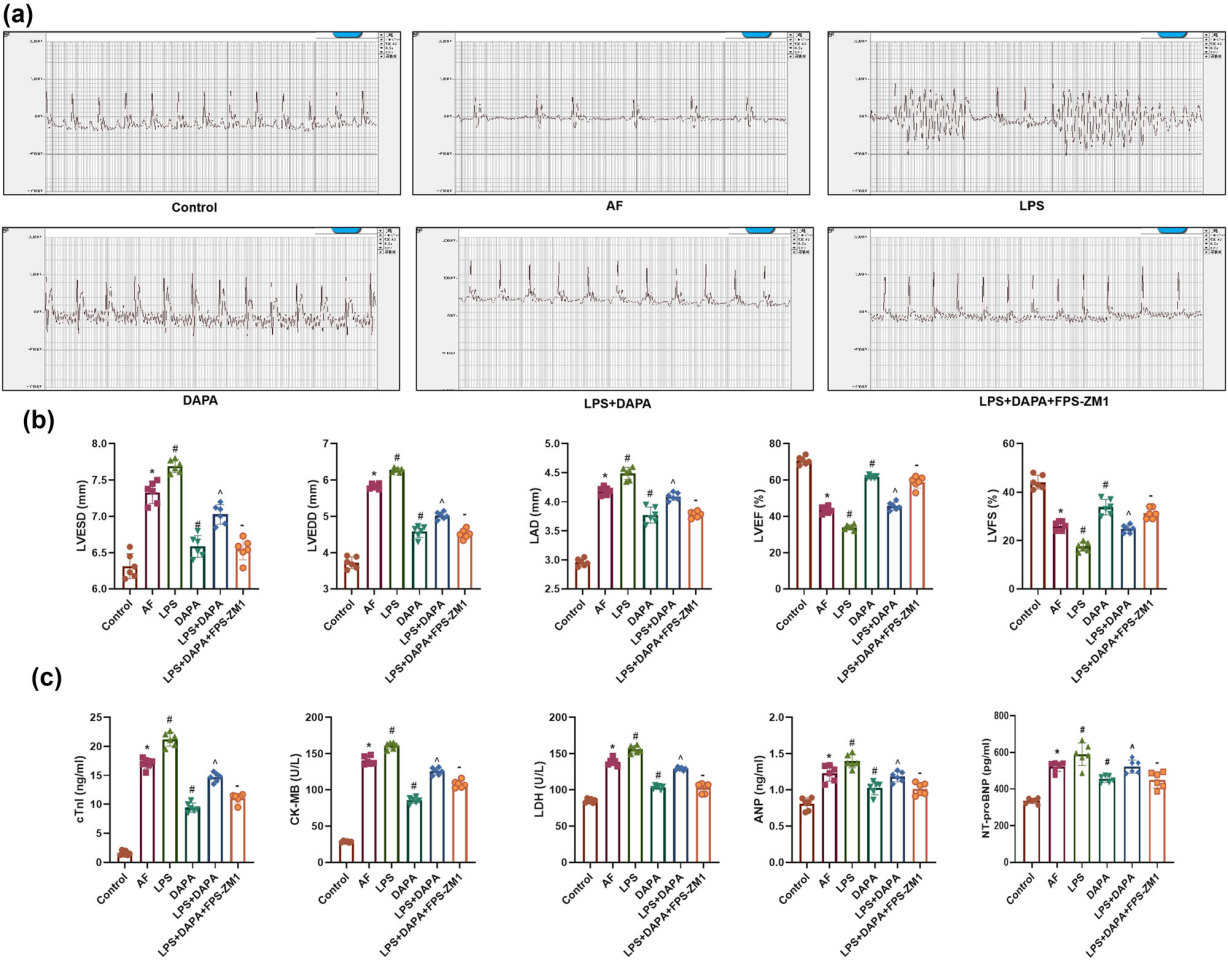
DAPA improves AF-induced cardiac dysfunction by inhibiting the HMGB1/RAGE pathway. (a) ECG assessing the degree of AF in rats. (b) Echocardiographic assessment of cardiac function indices, including LVESD, LVEDD, LAD, LVEF, and LVFS in left atrial tissues. (c) Biochemical analysis of myocardial injury markers (cTnI, CK-MB, LDH, ANP, NT-proBNP) in blood samples. *n* = 6. *p < 0.05 vs control. ^#^ *p* < 0.05 vs AF. ^*p* < 0.05 vs DAPA. – *p* < 0.05 vs LPS + DAPA.

ECG and echocardiographic data showed that AF rats developed significant arrhythmias, along with increased LVESD, LVEDD, and LAD, and reduced LVEF and LVFS (Figure 5a and b, *p* < 0.05). Arrhythmic changes and cardiac dysfunction were further exacerbated in the LPS group, as evidenced by more upregulated LVESD, LVEDD, and LAD as well as further downregulated LVEF and LVFS (*p* < 0.05). DAPA treatment partially restored cardiac function in the presence of LPS, while the addition of FPS-ZM1 to the LPS + DAPA group further enhanced cardiac function restoration (*p* < 0.05). Cardiac injury biomarkers (cTnI, CK-MB, ANP, NT-proBNP) were significantly elevated in the AF and LPS groups, indicating myocardial damage (Figure 5c, *p* < 0.05). DAPA significantly reduced these markers, although levels rose again in the LPS + DAPA group. Co-administration of FPS-ZM1 led to further reductions in injury markers, confirming enhanced cardioprotection via RAGE inhibition (*p* < 0.05).

### 3.5 DAPA mitigates AF-induced myocardial fibrosis through inhibition of the HMGB1/RAGE pathway

**Figure 6:**
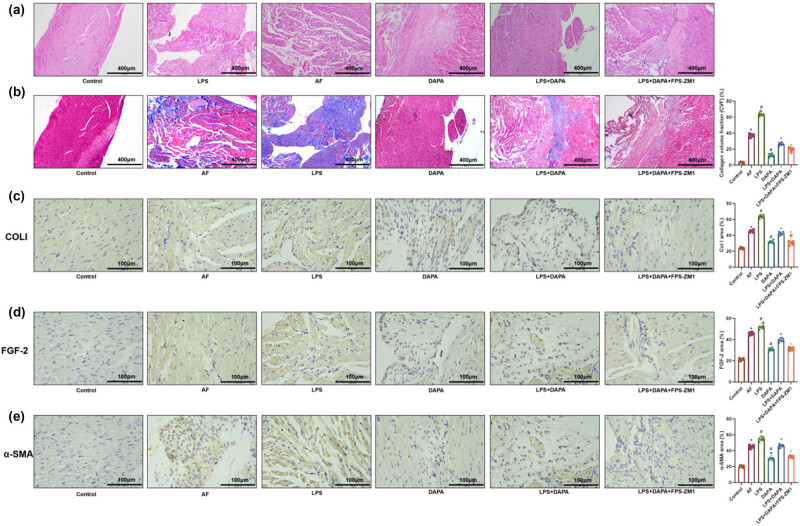
DAPA ameliorates AF-induced myocardial fibrosis by inhibiting the HMGB1/RAGE pathway. (a) HE staining for assessing pathological damage in left atrial tissues. (b) Masson’s trichrome staining assessing myocardial fibrosis in the left atrium. (c–e) IHC staining evaluating the expression of COL1, FGF-2, and α-SMA in left atrial tissues. *n* = 6. *p < 0.05 vs control. ^#^ *p* < 0.05 vs AF. ^*p* < 0.05 vs DAPA. – *p* < 0.05 vs LPS + DAPA.

Myocardial fibrosis was more severe in the AF group than in the control group, with increased interstitial cells, disordered myocardial arrangement, and higher expression of COL I, FGF-2, and α-SMA (Figure 6a, *p* < 0.05). Myocardial fibrosis was further worsened in the LPS group compared to the AF group, indicating HMGB1-mediated enhancement of fibrosis (Figure 6b, *p* < 0.05). In contrast, DAPA treatment reduced fibrosis markers, particularly in the DAPA-H group (Figure 6c, *p* < 0.05). In the LPS + DAPA group, fibrosis was partially mitigated by DAPA, despite ongoing HMGB1 activation (Figure 6d, *p* < 0.05). Additionally, FPS-ZM1 co-treatment further suppressed fibrosis markers, suggesting synergistic inhibition of HMGB1/RAGE signaling (Figure 6e, *p* < 0.05).

### 3.6 DAPA reduces AF-induced myocardial inflammation via suppression of the HMGB1/RAGE pathway

**Figure 7:**
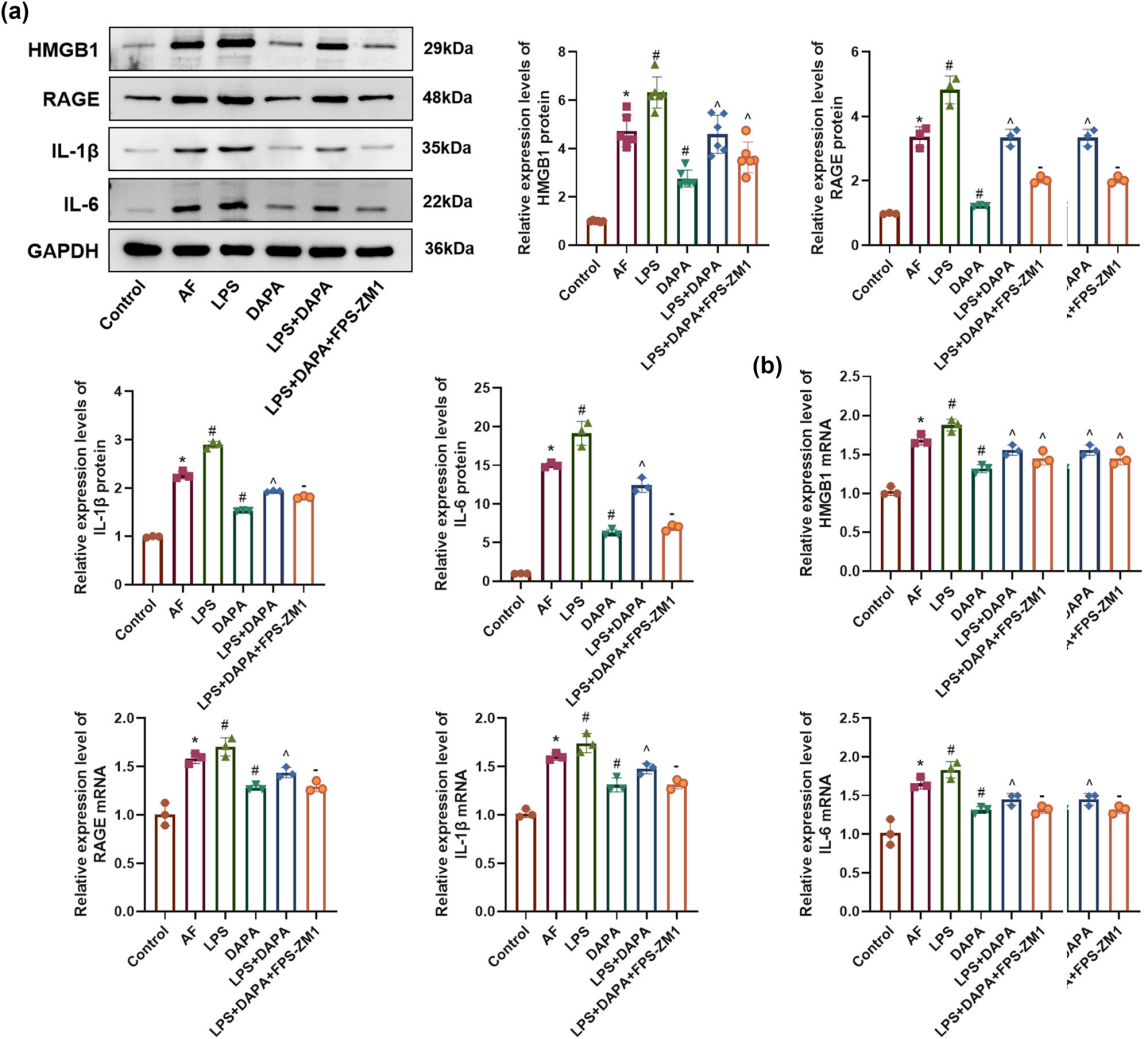
Effect of DAPA on myocardial inflammation in AF via the HMGB1/RAGE pathway. (a) and b) Expression levels of HMGB1, RAGE, IL-1β, and IL-6 in tissue measured by WB and RT-qPCR. *n* = 6. *p < 0.05 vs control. ^#^ *p* < 0.05 vs AF. ^*p* < 0.05 vs DAPA. – *p* < 0.05 vs LPS + DAPA.

The AF group exhibited significantly elevated levels of HMGB1, RAGE, IL-1β, and IL-6, confirming an inflammatory response (*p* < 0.05). These markers were further elevated in the LPS group (Figure 7a, *p* < 0.05). DAPA significantly suppressed the expression of HMGB1, RAGE, IL-1β, and IL-6, which suggests that DAPA inhibits the HMGB1/RAGE pathway and mitigates the inflammatory response induced by AF (Figure 7b, *p* < 0.05). In the LPS + DAPA group, inflammation persisted but was partially reduced by DAPA. Co-administration of FPS-ZM1 in the LPS + DAPA + FPS-ZM1 group led to further reductions, indicating enhanced anti-inflammatory effects through RAGE inhibition (*p* < 0.05).

## 4 Discussion

DAPA, a SGLT2 inhibitor, has emerged as a therapeutic agent of considerable interest due to its proven efficacy in diabetes and cardiovascular diseases [26,27]. However, its therapeutic potential and underlying mechanisms in AF remain insufficiently understood. To address this gap, we employed a rat model of AF to systematically evaluate DAPA’s effects on cardiac function, myocardial fibrosis, and inflammation, and found that DAPA exerts its cardioprotective actions in part by inhibiting the HMGB1/RAGE signaling pathway. These findings provide mechanistic insight and a potential translational framework for DAPA use in AF.

In our AF rat models, we observed significant cardiac dysfunction, characterized by increased LVESD, LVEDD, LAD, cTnT, CK-MB, ANP, and NT-proBNP, as well as decreased LVEF and LVFS, indicating marked aggravation of structural and functional remodeling. DAPA treatment significantly improved cardiac function in AF rats. ECG and echocardiographic analyses showed that high-dose DAPA (DAPA-H) reduced structural indices such as LVESD, LVEDD, and LAD, while restoring LVEF and LVFS. These results are consistent with studies demonstrating SGLT2 inhibitors’ protective effects in cardiovascular models independent of diabetes, such as ischemic cardiomyopathy, where benefits are attributed to improved myocardial energetics and reduced oxidative stress [28].

In terms of fibrosis, AF rats exhibited elevated CVF and increased expression of fibrotic markers such as COL1, FGF-2, and α-SMA. DAPA treatment reversed these fibrotic changes in a dose-dependent manner. Consistently, several studies have reported that DAPA can reduce the expression of cardiac inflammatory factors and fibrosis markers, with potential mechanisms involving the NLRP3 inflammasome and TGF-β/Smad pathways [29,30]. These findings also align with prior studies showing that SGLT2 inhibitors reduce ventricular fibrosis in pressure overload models [31], and our work extends this antifibrotic potential to AF. DAPA also demonstrated robust anti-inflammatory effects. It dose-dependently downregulated HMGB1, RAGE, and pro-inflammatory cytokines including IL-1β, TNF-α, and IL-6. HMGB1, a nuclear DAMP protein, translocates to the extracellular space under stress, activating RAGE and triggering NF-κB-mediated inflammatory signaling [32]. Moreover, HMGB1 remains persistently elevated in various cardiovascular diseases, including AF, and plays a particularly prominent role in atrial structural remodeling [8,33,34]. Our results suggested that DAPA’s anti-inflammatory action may involve interference with HMGB1-RAGE interaction, rather than HMGB1 suppression itself. This is supported by the finding that combined treatment with DAPA and FPS-ZM1 further reduced RAGE and cytokine levels without significantly altering HMGB1 expression. This aligns with structural studies proposing that SGLT2 inhibitors may hinder ligand-receptor interactions via steric or allosteric mechanisms [35,36,37], though this hypothesis warrants further molecular validation.

Although AMIO demonstrated greater efficacy in the direct improvement of certain cardiac functional parameters in our study, accumulating evidence highlights several unique advantages of DAPA in the management of patients with AF, particularly those with complex comorbidities. DAPA is generally well tolerated, and unlike AMIO, which is often limited by adverse effects such as thyroid dysfunction, pulmonary fibrosis, and hepatotoxicity DAPA is typically suitable for long-term use, especially in patients with heart failure or diabetes [38,39,40]. In addition, DAPA has been shown to reduce major cardiovascular events and provide renal protection in large clinical trials involving patients with chronic kidney disease and heart failure [41]. These broader benefits are rarely observed with traditional antiarrhythmic agents. Furthermore, recent studies suggest that DAPA and other SGLT2 inhibitors may exert antiarrhythmic effects via multi-pathway mechanisms, including the regulation of HMGB1/RAGE and TGF-β/Smad signaling [42,43], indicating integrated cardiovascular benefits beyond arrhythmia control alone. Nevertheless, it is important to acknowledge that, despite its relatively good safety profile, DAPA may be associated with adverse effects such as genitourinary infections and euglycemic diabetic ketoacidosis, particularly among susceptible populations [44,45]. These side effects are generally infrequent and manageable, but clinicians should remain vigilant and conduct individualized risk-benefit assessments when considering DAPA for AF patients. Further long-term and real-world studies are warranted to fully clarify the safety profile of SGLT2 inhibitors in diverse patient populations.

It should be noted that there is a current consensus that persistent activation of inflammation is a key driving force in the development of myocardial fibrosis. Our study also observed that inflammatory and fibrotic markers were simultaneously and significantly elevated in the AF group, indicating a close association between the two processes. However, the specific temporal and causal dynamics between inflammation and fibrosis in AF-associated fibrosis remain incompletely clarified. Some basic studies suggest that inflammation activation usually precedes collagen deposition, while the persistence of inflammation contributes to the progression of fibrosis [46,47]. As our study was based on data from a single time point, it could not dynamically illustrate the spatiotemporal evolution of the inflammation-fibrosis process. Therefore, future studies are recommended to employ multi-time-point tracking to more precisely elucidate their interactions.

## 5 Conclusion

This study demonstrated that DAPA treatment significantly reduced myocardial fibrosis, restored cardiac function, and inhibited the HMGB1/RAGE signaling activity in AF.

## References

[1] Saleh K, Haldar S. Atrial fibrillation: a contemporary update. Clin Med (Lond). 2023;23(5):437–41. 10.7861/clinmed.2023-23.5.Cardio2 PMC10541273 37775166

[2] Erhard N, Metzner A, Fink T. Late arrhythmia recurrence after atrial fibrillation ablation: incidence, mechanisms and clinical implications. Herzschr Elektrophysiol. 2022;33(1):71–6. 10.1007/s00399-021-00836-6 PMC8873127 35006336

[3] Bell A, Andrade JG, Macle L, Connelly KA, LaBine L, Singer AG. Approach to atrial fibrillation: Essentials for primary care. Can Fam Physician. 2023;69(4):245–56. 10.46747/cfp.6904245 PMC10112727 37072207

[4] Capucci A, Reiffel JA. Atrial fibrillation progression: another step in the RACE to full understanding. Europace. 2023;25(5):1–3. 10.1093/europace/euad071 PMC10227756 36967477

[5] Schnabel RB, Ameri P, Siller-Matula JM, Diemberger I, Gwechenberger M, Pecen L, et al. Outcomes of patients with atrial fibrillation on oral anticoagulation with and without heart failure: the ETNA-AF-Europe registry. Europace. 2023;25(9):1–12. 10.1093/europace/euad280 PMC10540669 37713182

[6] Uehara H, Gunji T. Differentiation between Takotsubo syndrome and coronary spastic angina in subjects undergoing catheter ablation for atrial fibrillation. J Arrhythm. 2023;39(5):838. 10.1002/joa3.12908 PMC10549833 37799791

[7] Benali K, Khairy P, Hammache N, Petzl A, Da Costa A, Verma A, et al. Procedure-related complications of catheter ablation for atrial fibrillation. J Am Coll Cardiol. 2023;81(21):2089–99. 10.1016/j.jacc.2023.03.418 37225362

[8] Karakasis P, Theofilis P, Vlachakis PK, Korantzopoulos P, Patoulias D, Antoniadis AP, et al. Atrial fibrosis in atrial fibrillation: mechanistic insights, diagnostic challenges, and emerging therapeutic targets. Int J Mol Sci. 2024;26(1):209. 10.3390/ijms26010209 PMC11720255 39796066

[9] Pozios I, Vouliotis AI, Dilaveris P, Tsioufis C. Electro-mechanical alterations in atrial fibrillation: Structural, electrical, and functional correlates. J Cardiovasc Dev Dis. 2023;10(4):149. 10.3390/jcdd10040149 PMC10141162 37103028

[10] Fu F, Pietropaolo M, Cui L, Pandit S, Li W, Tarnavski O, et al. Lack of authentic atrial fibrillation in commonly used murine atrial fibrillation models. PLoS One. 2022;17(1):e0256512. 10.1371/journal.pone.0256512 PMC8741011 34995278

[11] Zhu L, Wang Y, Zhao S, Lu M. Detection of myocardial fibrosis: Where we stand. Front Cardiovasc Med. 2022;9:926378. 10.3389/fcvm.2022.926378 PMC9557071 36247487

[12] Dhillon S. Dapagliflozin: A review in type 2 diabetes. Drugs. 2019;79(10):1135–46. 10.1007/s40265-019-01148-3 PMC6879440 31236801

[13] Arow M, Waldman M, Yadin D, Nudelman V, Shainberg A, Abraham NG, et al. Sodium-glucose cotransporter 2 inhibitor Dapagliflozin attenuates diabetic cardiomyopathy. Cardiovasc Diabetol. 2020;19(1):7. 10.1186/s12933-019-0980-4 PMC6953156 31924211

[14] Wu J, Liu T, Shi S, Fan Z, Hiram R, Xiong F, et al. Dapagliflozin reduces the vulnerability of rats with pulmonary arterial hypertension-induced right heart failure to ventricular arrhythmia by restoring calcium handling. Cardiovasc Diabetol. 2022;21(1):197. 10.1186/s12933-022-01614-5 PMC9516842 36171554

[15] Abd Elmaaboud MA, Estfanous RS, Atef A, Kabel AM, Alnemari KA, Naguib TM, et al. Dapagliflozin/hesperidin combination mitigates lipopolysaccharide-induced Alzheimer’s disease in rats. Pharmaceuticals (Basel). 2023;16(10). 10.3390/ph16101370 PMC10609711 37895841

[16] Yuan Y, Sun M, Jin Z, Zheng C, Ye H, Weng H. Dapagliflozin ameliorates diabetic renal injury through suppressing the self-perpetuating cycle of inflammation mediated by HMGB1 feedback signaling in the kidney. Eur J Pharmacol. 2023;943:175560. 10.1016/j.ejphar.2023.175560 36736941

[17] Arab HH, Al-Shorbagy MY, Saad MA. Activation of autophagy and suppression of apoptosis by dapagliflozin attenuates experimental inflammatory bowel disease in rats: Targeting AMPK/mTOR, HMGB1/RAGE and Nrf2/HO-1 pathways. Chem Biol Interact. 2021;335:109368. 10.1016/j.cbi.2021.109368 33412153

[18] Amornsupak K, Thongchot S, Thinyakul C, Box C, Hedayat S, Thuwajit P, et al. HMGB1 mediates invasion and PD-L1 expression through RAGE-PI3K/AKT signaling pathway in MDA-MB-231 breast cancer cells. BMC Cancer. 2022;22(1):578. 10.1186/s12885-022-09675-1 PMC9128129 35610613

[19] Su C, Jia S, Ma Z, Zhang H, Wei L, Liu H. HMGB1 promotes lymphangiogenesis through the activation of RAGE on M2 macrophages in laryngeal squamous cell carcinoma. Dis Markers. 2022;2022:4487435. 10.1155/2022/4487435 PMC8916867 35280439

[20] Zhang D, Wu C, Ba D, Wang N, Wang Y, Li X, et al. Ferroptosis contribute to neonicotinoid imidacloprid-evoked pyroptosis by activating the HMGB1-RAGE/TLR4-NF-kappaB signaling pathway. Ecotoxicol Environ Saf. 2023;253:114655. 10.1016/j.ecoenv.2023.114655 36812867

[21] Gkouveris I, Hadaya D, Elzakra N, Soundia A, Bezouglaia O, Dry SM, et al. Inhibition of HMGB1/RAGE signaling reduces the incidence of medication-related osteonecrosis of the jaw (MRONJ) in mice. J Bone Min Res. 2022;37(9):1775–86. 10.1002/jbmr.4637 PMC9474692 35711109

[22] Wang J, Zhang Q, Yao L, He T, Chen X, Su Y, et al. Modulating activity of PVN neurons prevents atrial fibrillation induced circulation dysfunction by electroacupuncture at BL15. Chin Med. 2023;18(1):135. 10.1186/s13020-023-00841-6 PMC10580609 37848944

[23] Zhao X, Liu Y, Han X, Wang X, Qu C, Liu X, et al. Dapagliflozin attenuates the vulnerability to atrial fibrillation in rats with lipopolysaccharide-induced myocardial injury. Int Immunopharmacol. 2023;125(Pt A):111038. 10.1016/j.intimp.2023.111038 38149574

[24] Parent S, Amant JS, Remortel SV, Kahn S, Vaka R, Courtman D, et al. Atrial fibrosis and inflammation in postoperative atrial fibrillation: Comparative effects of amiodarone, colchicine, or exosomes. JACC Clin Electrophysiol. 2024;10(6):1037–49. 10.1016/j.jacep.2024.02.019 38639701

[25] Wang G, Jin S, Huang W, Li Y, Wang J, Ling X, et al. LPS-induced macrophage HMGB1-loaded extracellular vesicles trigger hepatocyte pyroptosis by activating the NLRP3 inflammasome. Cell Death Discov. 2021;7(1):337. 10.1038/s41420-021-00729-0 PMC8572226 34743181

[26] Talha KM, Anker SD, Butler J. SGLT-2 inhibitors in heart failure: a review of current evidence. Int J Heart Fail. 2023;5(2):82–90. 10.36628/ijhf.2022.0030 PMC10172076 37180562

[27] Fu Q, Zhou L, Fan Y, Liu F, Fan Y, Zhang X, et al. Effect of SGLT-2 inhibitor, dapagliflozin, on left ventricular remodeling in patients with type 2 diabetes and HFrEF. BMC Cardiovasc Disord. 2023;23(1):544. 10.1186/s12872-023-03591-3 PMC10633988 37940879

[28] Jhund PS, Kondo T, Butt JH, Docherty KF, Claggett BL, Desai AS, et al. Dapagliflozin across the range of ejection fraction in patients with heart failure: a patient-level, pooled meta-analysis of DAPA-HF and DELIVER. Nat Med. 2022;28(9):1956–64. 10.1038/s41591-022-01971-4 PMC9499855 36030328

[29] Chen X, Yang Q, Bai W, Yao W, Liu L, Xing Y, et al. Dapagliflozin attenuates myocardial fibrosis by inhibiting the TGF-beta1/Smad signaling pathway in a normoglycemic rabbit model of chronic heart failure. Front Pharmacol. 2022;13:873108. 10.3389/fphar.2022.873108 PMC9136228 35645838

[30] Tian J, Zhang M, Suo M, Liu D, Wang X, Liu M, et al. Dapagliflozin alleviates cardiac fibrosis through suppressing EndMT and fibroblast activation via AMPKalpha/TGF-beta/Smad signalling in type 2 diabetic rats. J Cell Mol Med. 2021;25(16):7642–59. 10.1111/jcmm.16601 PMC8358881 34169635

[31] Chen X, Hocher CF, Shen L, Kramer BK, Hocher B. Reno- and cardioprotective molecular mechanisms of SGLT2 inhibitors beyond glycemic control: from bedside to bench. Am J Physiol Cell Physiol. 2023;325(3):C661–81. 10.1152/ajpcell.00177.2023 37519230

[32] Bangert A, Andrassy M, Muller AM, Bockstahler M, Fischer A, Volz CH, et al. Critical role of RAGE and HMGB1 in inflammatory heart disease. Proc Natl Acad Sci U S A. 2016;113(2):E155–64. 10.1073/pnas.1522288113 PMC4720305 26715748

[33] Wang B, Jiang T, Qi Y, Luo S, Xia Y, Lang B, et al. AGE-RAGE axis and cardiovascular diseases: pathophysiologic mechanisms and prospects for clinical applications. Cardiovasc Drugs Ther. 2024. 10.1007/s10557-024-07639-0. PMC12717123 39499399

[34] Wang X, Chen L, Wei J, Zheng H, Zhou N, Xu X, et al. The immune system in cardiovascular diseases: from basic mechanisms to therapeutic implications. Signal Transduct Target Ther. 2025;10(1):166. 10.1038/s41392-025-02220-z PMC12098830 40404619

[35] Rykova EY, Klimontov VV, Shmakova E, Korbut AI, Merkulova TI, Kzhyshkowska J. Anti-inflammatory effects of SGLT2 inhibitors: Focus on macrophages. Int J Mol Sci. 2025;26(4):1670. 10.3390/ijms26041670 PMC11854991 40004134

[36] Xu Y, Zhang C, Jiang K, Yang X, Chen F, Cheng Z, et al. Structural repurposing of SGLT2 inhibitor empagliflozin for strengthening anti-heart failure activity with lower glycosuria. Acta Pharm Sin B. 2023;13(4):1671–85. 10.1016/j.apsb.2022.08.023 PMC10149898 37139418

[37] Alsereidi FR, Khashim Z, Marzook H, Gupta A, Al-Rawi AM, Ramadan MM, et al. Targeting inflammatory signaling pathways with SGLT2 inhibitors: Insights into cardiovascular health and cardiac cell improvement. Curr Probl Cardiol. 2024;49(5):102524. 10.1016/j.cpcardiol.2024.102524 38492622

[38] Martinez FA, Serenelli M, Nicolau JC, Petrie MC, Chiang CE, Tereshchenko S, et al. Efficacy and safety of dapagliflozin in heart failure with reduced ejection fraction according to age: insights from DAPA-HF. Circulation. 2020;141(2):100–11. 10.1161/CIRCULATIONAHA.119.044133 31736328

[39] Vart P, Butt JH, Jongs N, Schechter M, Chertow GM, Wheeler DC, et al. Efficacy and safety of dapagliflozin in patients with chronic kidney disease across the spectrum of frailty. J Gerontol A Biol Sci Med Sci. 2024;79(2):1–10. 10.1093/gerona/glad181 PMC10809037 37527836

[40] You HS, Yoon JH, Cho SB, Choi YD, Kim YH, Choi W, et al. Amiodarone-induced multi-systemic toxicity involving the liver, lungs, thyroid, and eyes: a case report. Front Cardiovasc Med. 2022;9:839441. 10.3389/fcvm.2022.839441 PMC8918574 35295268

[41] Chertow GM, Correa-Rotter R, Vart P, Jongs N, McMurray JJV, Rossing P, et al. Effects of dapagliflozin in chronic kidney disease, with and without other cardiovascular medications: DAPA-CKD trial. J Am Heart Assoc. 2023;12(9):e028739. 10.1161/JAHA.122.028739 PMC10227210 37119064

[42] Duan HY, Barajas-Martinez H, Antzelevitch C, Hu D. The potential anti-arrhythmic effect of SGLT2 inhibitors. Cardiovasc Diabetol. 2024;23(1):252. 10.1186/s12933-024-02312-0 PMC11251349 39010053

[43] Rolski F, Maczewski M. Cardiac fibrosis: mechanistic discoveries linked to SGLT2 inhibitors. Pharmaceuticals (Basel). 2025;18(3):313. 10.3390/ph18030313 PMC11944955 40143092

[44] Zhou Z, Yao X. Safety assessment of dapagliflozin: Real-world adverse event analysis based on the FAERS database from 2012 to 2023. Heliyon. 2024;10(12):e33306. 10.1016/j.heliyon.2024.e33306 PMC11253505 39022025

[45] Gorgojo-Martinez JJ, Gorriz JL, Cebrian-Cuenca A, Castro Conde A, Velasco Arribas M. Clinical recommendations for managing genitourinary adverse effects in patients treated with SGLT-2 inhibitors: a multidisciplinary expert consensus. J Clin Med. 2024;13(21):6509. 10.3390/jcm13216509 PMC11546491 39518647

[46] Ariyama N, Aguero B, Valdes V, Berrios F, Bucarey S, Mor S, et al. Update of genetic diversity of porcine circovirus type 2 in Chile evidences the emergence of PCV2d genotype. Front Vet Sci. 2021;8:789491. 10.3389/fvets.2021.789491 PMC8718606 34977221

[47] Colpani V, Soares Falcetta F, Bacelo Bidinotto A, Kops NL, Falavigna M, Serpa Hammes L, et al. Prevalence of human papillomavirus (HPV) in Brazil: A systematic review and meta-analysis. PLoS One. 2020;15(2):e0229154. 10.1371/journal.pone.0229154 PMC7034815 32084177

